# Support or justice: a triangulated multi-focal view of sexual assault victim support in a UK sexual assault referral centre (SARC)

**DOI:** 10.1186/s13033-024-00631-z

**Published:** 2024-04-08

**Authors:** B. Kennath Widanaralalage, Anthony D. Murphy, Casey Loughlin

**Affiliations:** 1 King’s College London, London, UK; 2 University of Birmingham, Birmingham, UK; 3 University of Westminster, London, UK

**Keywords:** Sexual assault, Service provision, Barriers, Communication

## Abstract

**Background:**

Despite vast levels of underreporting, sexual assault remains an issue at scale in the UK, necessitating the presence of statutory and voluntary organisations in the support of victims. Understanding the experiences of all parties within this context is important for the resilience of support that can be provided at a systems level. This study examines the barriers faced by service providers when working with victims of sexual assault.

**Methods:**

Semi-structured interviews took place with eleven professionals working in or in conjunction with a Sexual Assault Referral Centre (SARC) in Southeast England, which were subsequently analysed using inductive thematic analysis.

**Results:**

Five themes were identified exploring SARC staff’s experiences with (i) communication breakdowns with external services; (ii) delivering support in an underfunded system; (iii) tailoring support to survivors’ needs; (iv) the Criminal Justice System fails victims of sexual assault; and (v) reckoning with burnouts and vicarious trauma.

**Conclusion:**

Significant gaps in UK service provision for sexual assault victims are identified, particularly within the criminal justice system, where legal and investigative processes are cited as retraumatizing. The results emphasize the urgency of enhanced training, coordination, resources, and trauma-informed practices across organizations to better serve victims and support overwhelmed providers. Prioritizing systemic improvements is crucial to address the complex needs of both victims and service professionals.

## Background

In the year ending March 2020, 773,000 adults were victims of sexual assault in England and Wales, with 618,000 females and 155,000 males reporting sexual victimisation within the last 12-months [1]. Only a fraction of victims (1 in 6 women and 1 in 5 men) report their assault to police [1]. The underreporting of sexual offences is concerning because victims face disproportionate long-term psychological, physical, and reproductive health consequences [2] as well as common mental health impacts like PTSD, depression, and anxiety [3]. One-third of rape victims will develop PTSD at some point in their lifetime and approximately 30% will experience serious depression [2]. Meanwhile, rape victims are three times more likely to develop a major depressive disorder, 13 times more likely to have attempted suicide, and are 26 times more likely to have two or more serious substance misuse issues, as well as a significant increased risk of self-harm and mental health service utilization [3].

Sexual Assault Referral Centres (SARCs), of which there are roughly 48 across the UK, act as a one-stop multidisciplinary medical unit providing services including medical, forensic, and psychological care [4–6]. SARCs integrate self-referrals, wider health systems, and services across the voluntary and criminal justice sectors. Critically, SARCs act as a point of referral for aftercare, including sexual health services, mental health services, safeguarding, counselling, and drug and alcohol services, accessible through the intervention of Police and SARC’s [6]. Indeed, 84% of referrals to SARCs in England are made by police [4]. Professionals report positive emotional experiences of working in SARC, including the meaningfulness of the work and the camaraderie among members of a team with shared values [7–10]. However, negative emotions from the experiences of this work and the emotional toll this takes are also noted [5, 11]. Crisis support workers identify systemic issues like lack of funding and resources, leading to insufficient training opportunities [14], a lack of awareness, and inadequate service of victims.

The experiences of personnel occur against a societal backdrop of normalized rape myths, which negatively shape people’s, systems’, and policies’ responses to victims [15]. Misconceptions around rape victims and offenders extend beyond the public [12, 13] to professionals in the criminal justice sector, such as police officers [14, 15]. The recent ‘Operation Soteria^1^’ highlight systemic issues in the police response to investigating rape and sexual violence in the UK [16]. These reports have emphasized the lack of specialist knowledge among police investigators about the nature of sexual offending and its impacts, disproportionate focus on testing victim credibility rather than investigating suspects, and the detrimental effects of high caseloads and under-resourcing on investigation quality and outcomes.

Given the importance of this work for justice and victim care, it is vital to understand SARC staff experiences, particularly around the perceived barriers and facilitators to providing quality services. However, research on Sexual Assault Referral Centres is relatively sparse. This research is vital to understand and improve the victim support process, occupational implications, and systemic care quality, which is imperative given NHS England’s emphasis on excellent rape victim services irrespective of age or gender [17]. A recent survey of Independent Sexual Violence Advisors (ISVAs) [18] found high caseloads, communication challenges with police, insufficient training and resources, and vicarious trauma were common. Recommendations included national training standards, supervision guidance, monitoring staff wellbeing, investigating maximum caseloads, and facilitating professional networking for training and support. The findings provide context about frontline staff challenges and needed systemic improvements to support victims.

## The present study

While limited research has examined the perspectives of ISVAs, less is known about the broader experiences of the diverse professionals operating within Sexual Assault Referral Centres (SARCs) in the UK. This study examined the experiences of professionals supporting victims and conducting investigations in collaboration with a UK SARC. Understanding the experiences of both victims and the professionals who support them is crucial to obtain effective, well-structured, and resourceful services, especially given the public health imperative. The present study takes a chronological, process-oriented approach to examine professionals’ perspectives on supporting victims of sexual assault from initial reporting through evidence gathering, accessing services, and liaising with police during investigations. Professionals were interviewed to gather insights into each stage of the process and difficulties faced to identify barriers for victims, service providers, and their relationship. The study examined organizational barriers, psychological challenges for victims and professionals, effective practices, and improvements needed across the victim support process from reporting to aftercare. The primary aim was understanding systemic challenges and potential ways to enhance services from the perspective of providers.

## Methods

### Participants

Eleven professionals were recruited from the author’s (CL) affiliation with a SARC in Southeast England. Convenience sampling was selected for recruitment as it relies on the principle that participants can be readily engaged at a low cost [19]. This approach is suitable for participant-led research, with CL’s established professional connections integral to the recruitment and collection of the data. The constitution of this sample is reflected in Table 1. Participants consisted of eleven females aged between 27 and 66 years old, all with at least two years’ experience in their job role. This multi-focal sampling allowed for this study to examine the SARC as a healthcare system, taking a triangulated approach to data collection and analysis, placing the experiences of SARCs and those they serve as the central issue. All participants had some form of training before beginning their roles in relation to sexual offences, ranging from on-the-job training, shadowing existing staff, online training, and medical and forensic training. All participants identified as female and White British, with participants reporting extensive experience working with sexual violence victims, with cases ranging from 60 to several thousands.

**Table 1:** Demographic information of SARC staff participants

	*N* = 11
**Age**	
18–30 years old	1
30–50 years old	4
> 50 years old	6
**Sex**	
Female	11
**Ethnicity**	
White	11
**Role**	
Manager	1
Forensic Nurse Examiner	2
Crisis Worker	3
Sexual Offences Examiner	1
Forensic Medical Examiner	1
ISVA	3
Sexual Trauma Counsellor	1
**Years working in the role**	
2	2
3–5	5
5–10	2
> 10	2
**Number of cases worked on**	
60–100	2
100–300	4
300–1000	3
> 1000	2

### Procedure and interviews

After obtaining ethical approval from [anonymised for peer review], the researcher contacted eligible participants via email. Participants were provided information and gave informed consent. Interviews were conducted virtually using a meeting platform and audio recorded for later transcription. Before starting, the interviewer explained the study’s purposes and objectives and participants’ rights to anonymity, confidentiality, and withdrawal. Throughout the interview, the interviewer verified participant comfort and ensured they were not distressed and willing to continue before asking questions. Interviews lasted 35–45 min. Participants were debriefed after completion. A sensitivity/distress protocol was followed to prioritize wellbeing and identify signs of distress. The interview questions focused on triangulating experiences, beliefs, and perceptions across SARC professions. Questions centred on job roles, training, areas for development, the victim support process, what works well, needed service improvements, and participants’ perceptions and experiences of their SARC’s processes. Additional discussion areas included experiences with victim blaming, shame, conviction rates and their impacts, difficulties working with victims, and perceptions of victim experiences across the support process from initial reporting through aftercare. After interviews were transcribed, inductive thematic analysis [20] was conducted to identify themes related to the research question. Thematic analysis provided appropriate flexibility for the multi-focal sample without imposing strict epistemological stances.

### Rigour in data analysis

The multifocal sample required triangulating differing perspectives. Investigator triangulation was used with multiple researchers generating a range of perspectives on the date [21]. The interviews were conducted by the third author (CL), who worked at the centre. To avoid biases associated with this insider position, all authors collaboratively developed a strong interview schedule using a participant-researcher methodology, with the interviewer having an ‘inside’ perspective [22]. However, the two other authors (BKW, AM) supervised data collection and conducted analysis to ensure objectivity and neutrality were maintained. Their involvement at all stages safeguarded against undue influences from the interviewer’s existing relationships with participants. The researchers’ diverse backgrounds and experienced further enhanced the multi-perspective approach, facilitating a rich examination of the data. High levels of congruence were established in this study.

## Results

**Fig. 1:**
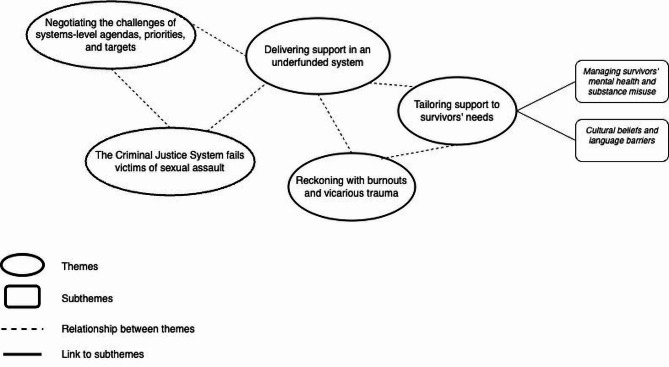
Thematic map of barriers for providing support in Sexual Assault Referral Centres

Five themes were identified describing perceived barriers for providing adequate support: (i) negotiating the challenges of systems-level agendas, priorities, and targets; (ii) delivering support in an underfunded system; (iii) tailoring support to survivors’ needs; (iv) the Criminal Justice System fails victims of sexual assault; and (v) reckoning with burnouts and vicarious trauma. To illustrate the interconnectedness of the themes, a thematic map is provided (Fig. 1).

### Theme 1: negotiating the challenges of systems-level agendas, priorities, and targets

Dealing with services with different agendas (e.g. police officers, ISVAs) inevitably resulted in miscommunication and attrition between practitioners. Participants expressed concerns around obtaining support for survivors in mental and social health care sectors, given the strict eligibility criteria for support. Participants were frustrated with how external services took a ‘checklist’ approach to mental health support provision, setting seemingly arbitrary thresholds for who could receive mental health care. The notion of “a constant fight” dominated participants’ accounts, with SARC staff feeling undervalued by other services for their contributions to supporting and safeguarding survivors.

Frustrations were often reflected in encounters with police officers. Differing agendas between the two services were described at length, with participants observing how officers’ limited knowledge of SARC procedures directly affected survivors’ wellbeing. Participants described survivors arriving at SARCs misinformed and confused for their first forensic examinations after interacting with police. Participants noted how officers may inadvertently misinform survivors, creating unnecessary distress and confusion. Some argued that officers’ inexperience was detrimental to the collection of forensic samples, hindering the progress of the investigation. A Sexual Offences Examiner in this study highlighted how officers’ limited understanding of SARC processes was exemplified by their focus on evidence collection/retrieval over survivors’ psychological wellbeing – somewhat in contrast with SARC’s approach to deliver on these important objectives (see Table 2).

**Table 2:** Overview of Theme 1 with representative quotes

Theme	Description	Quotes
Negotiating the challenges of systems-level agendas, priorities, and targets	Challenges in sexual violence investigations and support provision, highlighting issues like miscommunication, confidentiality concerns, and difficulties in obtaining support for survivors in mental and social health care sectors.	‘Mental health services have their thresholds to meet before somebody gets support and the same with adult social care. Quite often, it’s hard to reach those thresholds and you do find yourself in situation where you feel support for a person is necessary, but they don’t tick enough boxes to receive that which is frustrating and a constant fight.’ (Independent Sexual Violence Advisor) ‘It can be frustrating from the crisis worker side … police officers take their time, and they sometimes get lost and don’t necessarily know what they’re doing.’ (Crisis Worker) ‘…the client will say [that] they have been told it’s okay to wash or things like that.’ (Crisis Worker) ‘ Obviously, we have time constraints but there’s always a little bit of flexibility, so we don’t have to see them at 3 in the morning, we can see them when they’ve slept, if they’re in hospital we can wait till they have been discharged and rested before they come to see us.’ (Sexual Offences Examiner)

### Theme 2: delivering support in an underfunded system

The consequences of limited funding and resources were observed across several “moments” of service provision. Participants noted that insufficient resources contributed to a shortage of trained police officers, particularly in sexual offenses. This linked to communication gaps on policies for bringing sexual offense victims to the SARC. The consensus was that more officers trained in sexual offenses would greatly improve victim support. ISVA participants highlighted the interconnectedness of services involved in supporting victims and, simultaneously, the competing interests and priorities: delivering post-abuse care against conducting rapid and effective investigations. Participants emphasised the cascading effect of limited resources on SARCs, ISVAs, and police officers, signalling less training and less staff available in all these systems.

Participants observed how their workload had substantially increased at the detriment of the time they could allocate to survivors. In many ways, participants’ views on the lack of resources reflected a dissatisfaction towards the level of support and influence they could have in survivors’ post-abuse experiences with the services. In reporting their views on the current state of service provision for survivors of sexual violence, participants’ frustration became evident. Throughout the interviews (see Table 3), participants emphasised the need for a more nuance in service provision, recognising that mental health support goes beyond strict use of “toolkits” to tailor support to survivors’ unique needs and demands.

**Table 3:** Overview of Theme 1 with representative quotes

Theme	Description	Quotes
Delivering support in an underfunded system	Limited funding for sexual violence support services results in a shortage of trained police officers, communication gaps, and increased workload for SARCs and ISVAs. Participants express frustration with compromised victim support, emphasizing the need for more nuanced, tailored mental health services beyond rigid toolkits.	‘There needs to be more community mental health on the NHS, there is a gap there as well. I think sexual trauma there really is not, there is a gap in those services. They are very stretched, and I understand that.’ (Sexual Trauma Counsellor) ‘There aren’t enough resources, not enough police officers, not enough ISVA’s, probably not enough staff in all these different sectors that are able to give victims or survivors the time that they deserve. As an ISVA our caseloads are very high at the moment, so we do not have the capacity to do the work that we would like to do. I’m sure it’s the same for the police as well. When someone first discloses a sexual crime, first responders are typically the neighbourhood policing teams [and] they’ve got very limited training with dealing with sexual violence […] that first contact when someone discloses is key - if you don’t get it right, whether it is the police officer, us [ISVAs], the SARC … whoever takes that first disclosure, if the language isn’t right, if the body language isn’t right … it can have a massive impact, from reporting through to aftercare.’ (Independent Sexual Violence Advisor) ‘So, I suppose what I find difficult is not having the time to be with every single one of my clients. Because I think if we had more time to do that then we would be able to certainly help with having a bigger impact and helping them to move forward. So, I think that’s maybe what I find more difficult, is not having enough time really, to do what I’d like to be able to do.’ (Independent Sexual Violence Advisor) ‘People are being encouraged by services to come forward and ask for help, but the structure isn’t there to give them the help they need and trauma therapy, one size doesn’t fit all, a cropped version of therapy doesn’t fit all. So, this is where in my opinion a lot of the improvements needs to be looked at.’ (Independent Sexual Violence Advisor)

### Theme 3: tailoring support to survivors’ needs

Participants highlighted the importance of rapport building, forensic examinations, and effective communication, which often depended on how victims presented and their circumstances. The unpredictable nature of the work was emphasized, with survivors’ unique needs posing challenges. Two subthemes were identified: (**A**)*cultural beliefs and language barriers*, and (**B**)*managing survivors’ mental health and substance misuse issues.*

### Subtheme A: Cultural beliefs and language barriers

Participants highlighted cultural beliefs around intimate partner relationships outside of marriage causing feelings of shame for survivors with strong religious backgrounds or from minoritized ethnicities. Survivors’ unique needs created pressures for providers to deliver care and gather evidence as sensitively as possible. Gathering survivors whose first language is not English was particularly challenging in terms of establishing effective communication, whilst avoiding exacerbating survivors’ feelings of shame and guilt [23–27]. Cultural and language barriers were especially apparent when interpreters were needed to establish a line of communication with survivors. An extract from a Forensic Nurse Examiner (see Table 4a) highlighted how cultural barriers place survivors from minority ethnicities in seemingly “impossible” situations, such as disclosing details of a (non-consensual) sexual interaction to a man. Discussions on intimate and/or sexual relations are often stigmatised and silenced within minoritized ethnicities [28], thus placing SARC staff members in a delicate position of delivering support whilst respecting the cultural beliefs and needs of every survivor.

**Table 4:** a: Overview of Theme 3, Subtheme A, with representative quotes

Theme		
Tailoring support to survivors’ needs		
**Subtheme A** Cultural beliefs and language barriers	Cultural beliefs, language barriers, and shame pose challenges during forensic examinations. SARC staff navigate the delicate task of supporting survivors, respecting diverse cultural beliefs, and addressing language difficulties for effective communication.	‘If they’re religious and they’ve been touched by someone other than their partner. Feelings of being degraded. Cultural views if they’ve, different cultures have different views on sexual assault or rape, that they’ve been spoiled, they’ll never marry a man or women.’ (Forensic Nurse Examiner) ‘I think like basic things like language and culture differences can make examinations really complicated also like one of the really difficult things is being able to get the information you need to provide the examination service that is required without retraumatising them and getting them to go into all this details about what’s happened to them which has probably already been asked by officers multiple times by the time they come and I think one of the hardest things is knowing, being able to get the information you need to support them without making them feel worse, I think that’s really hard. Sometimes you get it wrong, and you notice you’ve got it wrong, and it feels terrible and other times they seem okay and its fine’ (Forensic Medical Examiner) ‘Language barriers are difficult, especially with one case that springs to mind, there was a male interpreter for a religion that doesn’t talk about intimate topics with men unless the man is their husband and even still, I imagine that probably doesn’t happen very often’ (Forensic Nurse Examiner)

### Subtheme B: managing survivors’ mental health and substance misuse issues

Participants observed that survivors frequently arrived at the centre with ongoing mental health and substance misuse issues. Examples in Table 4 illustrated how this impacted service provision and the survivors’ capacity and willingness to engage. The challenges underscored the demands and flexibility required by SARC staff when dealing with survivors of sexual violence. Survivors’ ability and willingness to engage with support services are influenced by the nature of their experiences and personal backgrounds. As such, substance use and mental health issues notably affect individuals’ meaningful engagement with healthcare services. SARC staff must address not only the immediate needs of survivors’ sexual trauma but also any regression in their overall well-being. Importantly, providers experienced a sense of isolation from other stakeholders. This is because several survivors approach the service before engaging with the Criminal Justice System, placing significant responsibility and even safety concerns on SARCs and their staff. Understandably, participants expressed frustration at their inability to deliver adequate care, often directing their concerns towards the Criminal Justice System.

**Table 4:** b: Overview of Theme 3, Subtheme B, with representative quotes

Theme		
Tailoring support to survivors’ needs		
**Subtheme B** Managing survivors’ mental health and substance misuse issues	Survivors arriving at the center with mental health and substance issues impact SARC service provision. Staff face challenges engaging with survivors, dealing with overall well-being issues, and feeling isolated from other stakeholders.	‘So, we have people who are um addicted alcohol or drugs and its actually assessing them whether they have capacity to go through with the examination or whether we delay slightly, we see them and discuss the different options.’ (SARC manager) ‘There have been occasions whereas well where a victim has gotten aggressive and threatened to assault a member of the staff there’s people with quite serious mental health issues um yeah and if from things like you know if a client comes in as a self-referral, they haven’t got police with them, we tend you know like kick off or something we haven’t got back up from the police.’ (Crisis Worker) ‘I suppose if there still under the influence of any alcohol or drugs, it can be quite difficult to you know get a rapport with someone.’ (Crisis Worker)

### Theme 4: the Criminal Justice System fails victims of sexual assault

Participants consistently expressed frustration as service providers regarding the lengthy police investigations, emphasizing the negative impact on both SARCs and the police service’s credibility. Their primary concern, however, was the adverse effect of policing on survivors’ well-being and confidence in service provision. Participants acknowledged the challenges faced by police officers in investigating sexual offenses but noted the lasting impact on their clients. The overwhelmed state of the police service often left survivors in limbo for extended periods, with minimal hope for a successful outcome. The issues extended beyond procedural matters to expertise, with officers overlooking the nuances of sexual offenses and survivors’ mental health needs.

Participants frequently raised concerns about low conviction rates for perpetrators of sexual offenses, expressing disappointment, frustration, and anger. They attributed these low rates to insufficient funding and resources for investigations. Some participants were so dissatisfied with the criminal justice system that they questioned whether to encourage survivors to report to the police. A suggested solution was additional training for police officers in handling sexual offenses, including awareness of the impact of sexual trauma and understanding SARC protocols.

The extracts in Table 5 highlight how officers’ lack of training leads to overlooking the unique needs of sexual violence survivors. Failure to recognize the gendered nature of survivors’ experiences and insensitivity towards their need for privacy after reporting were cited as examples. Participants emphasized how this lack of training affected survivors’ well-being up to the point of handover, leaving SARCs to address the aftermath. The main recommendation from participants was an increased focus on specialized training, with praise for the role and expertise of specially trained officers.

**Table 5:** Overview of Theme 4 with representative quotes

Theme	Description	Quotes
The Criminal Justice System fails victims of sexual assault	Service providers express frustration with lengthy police investigations, citing detrimental effects on SARCs’ and police credibility. Concerns include survivors’ well-being, communication issues, and the need for additional training to address survivors’ unique needs.	‘We’re talking minimum 18 months to 2 years, it’s too long for the individual, it retraumatises victims, its completely wrong and that really needs to be looked at and escalated to a much quicker process.’ (SARC manager) ‘If a client knew that, even if a trial didn’t happen for another two years actually they could get their head around that but it’s this being in a limbo for up to a year of not knowing whether the charge is going to go forward’ (Sexual Trauma Counsellor) ‘The more you do this type of work it gets increasingly difficult to not say to them quite overtly don’t bother reporting because it’s only going to retraumatise you, it’s going to result in nothing anyway so don’t put yourself through that, concentrate on trying to heal.’ (Crisis Worker) ‘Dealing with the police officers that may be bringing a victim of sexual assault to us who don’t understand the process and then that’s quite difficult um or can be quite difficult because they don’t always know what the journey is like for the victim either’ (Crisis Worker) ‘I think especially male police officers bringing in a female client and vice versa really, I think it can be fairly inappropriate, just not so good for the client.’ (Crisis Worker) ‘I would say it should be from a police point of view it should be sexual violence officers and people that are trained to deal with that. Just for a better experience for the client and for the organisation of everything and having what you need with you for the journey for the client. In [removed for anonymity] you do get sexual violence officers start to finish.’ (Forensic Nurse Examiner)

### Theme 5: reckoning with burnouts and vicarious trauma

Service providers described their work as rewarding, but alongside the satisfaction, participants acknowledged significant emotional challenges. Reflecting on specific cases, they noted the lasting impact on their personal lives. Participants stressed that managing others’ trauma heightened the risk of vicarious trauma, underscoring the importance of self-care and seeking help. The accounts revealed a dual notion of self-care, with some aiming to separate personal and professional lives, recognizing the toll of dealing with others’ trauma. Balancing personal needs while ensuring successful support delivery was a crucial process in how participants approached their work. Several extracts emphasized this dual self-care approach as an act of self-preservation, guarding against both immediate emotional impacts and recurring frustrations. Participants expressed a need to shield themselves from disappointments and frustrations experienced by survivors, highlighting their emotional investment in clients’ psychological recovery and service experience.

**Table 6:** Overview of Theme 5 with representative quotes

Theme	Description	Quotes	
Reckoning with burnouts and vicarious trauma	Services face emotional challenges when working with sexual assault survivors. Participants stress the dual notion of self-care, aiming to separate personal and professional lives to protect against the emotional impact of survivors’ trauma and recurring frustrations in their work.	‘My overall experience is one of quite overwhelming at the number of vulnerable women and men out there that find themselves in that situation of being subject to a sexual assault. I do get overwhelmed by it’ (Crisis Worker) ‘I’ve recently had two bereavements so I must keep myself well supported because dealing with trauma it has an impact on myself so that is something I have to be aware of that I am not taking on vicarious trauma. So that I need to be well supported.’ (Sexual Trauma Counsellor) ‘It’s quite difficult, sometimes there are cases that you find hard to stop thinking about when you go home, ones that you know resonate with you longer than others. Just frustration at not always being able to offer the services we’d like to.’ (Sexual Offences Examiner)	

## Discussion

The present study enhances the understanding of the barriers for service provision within Sexual Assault Referral Centre’s in the UK using a qualitative approach. Results revealed several barriers experienced by service provision and the consequences for both professionals and victims of sexual assault. Recent research with ISVAs highlight similar challenges (high caseloads, communication issues with external services, insufficient training and resources, and vicarious trauma) [18], with recommendations focusing on systemic improvements needed to support staff working with victims of trauma. Our findings expand on this literature by providing a descriptive and experiential account of overlapping barriers and issues encountered by SARC service providers, with procedural (themes 1 and 2) and unique victim- characteristics (theme 3) shaping several negative outcomes, both in terms of successful criminal investigations (theme 4) and personal mental health consequences for service providers (theme 5).

Issues with communication and victim support are known in the Criminal Justice System [30–33]. This study illustrated additional communication challenges arising from competing agendas and limited understanding across voluntary and criminal justice systems. As victims typically report complex psychological and physical needs [34–37], including post-traumatic stress and suicidality [38], requiring careful management from SARC centres as they serve as the reporting and support access point [5, 11]. Staff reported experiencing pressures around balancing patient care and supporting police investigation to collect forensic evidence within a limited timeframe [39]. The SARC-police interactions documented in this study raise concerns around officers’ unawareness on SARC processes and procedures, especially when more than 80% of referrals to SARCs in England are through local police [4]. The study illuminates the adverse impact of conflicting agendas among service providers on both the mental health of patients and the prosecution of sexual offenses. By underscoring the confusion and misinformation experienced by survivors in their interactions with the police, the research exposes how breakdowns in communication between components of the system can exacerbate survivors’ trauma.

Insufficient funding for sexual assault support, a well-documented barrier in the UK’s third sector [30, 31, 40–43], persists despite recent pledges to increase resources [44]. Studies consistently reveal disproportionate resource allocation, creating a ‘post-code lottery’ for aftercare [6, 29, 45–47]. Our study echoes these findings, with participants describing their experiences in an underfunded and occasionally dysfunctional system. Providers noted ‘gaps’ in victim support, including a lack of specialization in related sectors (policing, ISVA, community mental health), contributing to recurrent communication breakdowns. The study highlights the ripple effects of an increasing number of sexual assault survivors seeking immediate help [42], emphasizing the third sector’s inability to cater to unique needs and the interconnectedness of victim support services. Crucially, underfunding is identified as both an organizational and systemic barrier [48], hindering staff training and specialization across sectors and impacting current staff’s ability to meet patients’ unique demands.

Our findings align with recent police inspection reports, like Operation Soteria Bluestone [16], highlighting issues such as investigators’ lack of specialized knowledge, disproportionate emphasis on victim credibility, and the adverse effects of under-resourcing and high caseloads. SARC staff experiences in our study mirror these identified problems in police responses to sexual assault, emphasizing the urgent need for collaborative initiatives to enhance training, allocate resources, improve inter-agency coordination, and overall service provision for victims. This study also offers one of the first accounts of SARC engagement with ethnic minority survivors, revealing challenges related to effective communication and navigating cultural dynamics in understanding and discussing sexual violence within these communities. Limited UK knowledge on minority experiences of sexual violence exists, with some studies suggesting unique cultural pressures leading survivors to conceal trauma [49, 50]. Our study explores these barriers through the perspectives of SARC staff, whose roles pose challenges in encouraging disclosure of survivors’ experiences [2, 11]. The analysis underscores the “impossible” position faced by survivors reluctant to disclose to male interpreters and providers striving to maintain a high-quality service and gather crucial forensic evidence [2, 11].

Participants detailed the simultaneous challenges faced by sexual assault survivors upon reaching a SARC, reflecting evidence on complex outcomes associated with rape victimisation, where survivors immediately report anxiety, shame, post-traumatic stress, and suicidality [51]. Similarly, substance use in sexual assault survivors is an established finding in the literature [35, 52]. Furthermore, evidence from Brooker and Durmaz [2] revealed that 40% of SARC clients are known to local mental health services, yet almost two-thirds of services report problems referring clients for further community mental health support. It is of interest that SARC staff in this study emphasised their concerns around their ability to engage and support survivors with on-going mental health and substance abuse issues. Such findings reflect other research in SARCs, where staff members reported feeling overwhelmed by the volume of work received and the number of overlapping roles they undertook, from providing mental health support to facilitating sexual assault investigation [11].

SARC staff stressed how their work was shaped by a close collaborations with criminal justice partners [4], raising several concerns identified in past research calling for meaningful reforms to the policing of sexual assault in the UK [30–32, 53]. Among the challenges, SARC staff emphasised the impact of lengthy and unsuccessful criminal investigation on survivors, with some data suggesting that sexual offences receive an investigative outcome after more than 77 days against an average time of 11 days for other types of offences [54]. Upon entering the Criminal Justice System, survivors of sexual violence are often “stuck” in complex and extensive investigative procedures, with significant mental health impacts [30, 53]. Such effects are exacerbated by the recognised low conviction rates in the UK [54], with providers showing a profound disappointment and sense of hopelessness and helplessness towards their role in facilitating police investigation. Underpinning participants’ views towards the Criminal Justice System was an awareness of the need for reform, both in training and specialism, that embedded trauma-informed care in every step of police investigation [55, 56].

Theme 5 best captured the extent to which working in a dysfunctional system impacts on providers’ mental health. Findings from this study supports previous research, with SARC staff members consistently reporting important emotional consequences in working with sexual assault survivors. Recent evidence from Horvarth et al. [5] provides a detailed profile of the emotional demands to SARC staff, which include flexibility and resilience to reduce vicarious trauma. The present findings enhance these notions by providing an experiential account of what participants described as “managing others’ trauma”, a process of carefully providing mental health support during the referral and examination processes aimed at safeguarding survivors’ wellbeing, whilst maintain professional boundaries. Such a notion is not uncommon in medical and mental health systems [57], with healthcare practitioners often having to balance patients’ needs against their own in order to meet the many demands of their role, whilst promoting patients’ autonomy [58].

Autonomy and empowerment are core principles in trauma informed care [59] and emerged as fundamental in how SARC staff members viewed their roles and duties towards sexual assault survivors. Findings from this study also extend our knowledge on the impacts on SARC staff by detailing providers’ deep emotional involvement in delivering care and support police investigations, thus sharing the disappointments and frustrations experienced by their clients. Indeed, earlier research by Martin [9] reports how feelings of powerlessness and helplessness towards the inadequate response from collaborating systems, including criminal justice services, are detrimental to frontline workers’ ability to operate and support victims. Throughout our analysis, SARC staff often expressed a sense of disillusionment in their role, at times even suggesting that perhaps they would encourage survivors to engage the service to seek support rather than seeking justice.

### Implications and recommendations

By viewing SARCs at the system level and examining the experiences, challenges, and barriers from a multi-focal perspective, this study contributes to discussions among commissioning bodies and those in managerial positions of the discrepancies within the system and may inform service enhancement efforts at multiple levels, considering both practical implications at a macro level (e.g., communication with partner organisations) and at a micro level (e.g., mental health and wellbeing of practitioners). Other service providers who operate within the nexus of work done by SARCs (e.g., police, mental health services) may benefit from more resources and training to address the needs of sexual violence victims to drive collective improvement efforts. This is particularly important given the necessarily collaborative nature of the work done, where communication, information sharing, criteria and thresholds, and agency policies and procedures may all impact the efficiency and effectiveness of the work being done.

### Limitations of this study

Despite making a significant contribution to the literature, this study is not without limitation. Each participant was female and working for or in conjunction with the same Sexual Assault Referral Centre in the southeast of England. The recent national survey of ISVAs by Horvath and colleagues [20] highlighted limitations related to demographic homogeneity and regional variability in service provision and caseloads. While our in-depth qualitative approach provides insight into staff experiences in a high-volume SARC, incorporating a larger and more diverse sample could strengthen transferability and generalisability. In particular, the national surveys capture a broader demographic profile and wide regional distribution across England, Wales, and Northern Ireland. It is therefore important to consider limitations and sampling approaches of the present study to further enhance the rigor of our methodology and contextualize our findings within national trends. Finally, it is important to note that all participants were female and working within a service that caters for a disproportionately female service-user base. This may have influenced participants’ experiences, beliefs, and attitudes related to sexual violence, those who are victims, and those who perpetrate it. Comparing these findings with recent work with service providers in the UK supporting male victims of sexual violence [28] highlights the nuances and uniqueness of the populations who are victims of this crime, and the divergences and convergences in front-line workers’ support experiences.

It is important to note that one member of the research team was in a professional working relationship with most of the participants. While this is primarily seen as a benefit in engaging in user-led research, enhancing the rapport between researcher, and researched, there is reason to acknowledge this as having a potential impact on interview and analysis. A close working relationship with the remaining members of the research team and consistent reflective dialogue helped to minimise the impact this would have on the data and subsequent analyses.

Our entirely White British sample raises valid questions about staff training in cultural competency and the need for more a diverse staffing base, especially given participants’ accounts of challenges responding to minority survivors. While participants endeavoured to provide culturally sensitive care, the lack of lived experience and insider perspectives among staff may result in cultural gaps in understanding or cultural assumptions. Ensuring staff receive robust diversity training alongside efforts to recruit more diverse SARCs teams could enrich capacity for culturally sensitive and tailored support. As highlighted in existing research, pressure to conceal abuse for fear of stigma exists in some minority communities [49, 60]. Hiring staff with insider knowledge of specific cultural barriers could aid engagement with reluctant survivors. More diverse teams may be better equipped to understand nuanced needs of minority groups and modify services accordingly while avoiding internalized assumptions.

## Conclusion

The study revealed multifaceted barriers faced by UK SARC staff supporting sexual assault survivors, including communication breakdowns, resource constraints, meeting diverse needs, justice system frustrations, and vicarious trauma. The findings underscore the critical need for systemic improvements through enhanced inter-agency coordination, increased funding and resources, cultural awareness, trauma-informed reforms, and staff wellbeing initiatives. The study provides valuable insights into frontline experiences to inform organizational change and policy responses to sexual violence. Further research with larger, more diverse samples and perspectives is needed to build on these important findings. Overall, a holistic approach focused on collaboration and survivor-centered care is essential to empower both victims and support providers.

## Data Availability

Due to concerns regarding individual privacy and confidentiality, the datasets generated and/or analysed during the current study are not available for public access.

## Footnotes

1 Operation Soteria Bluestone is a collaborative program between police forces and academics in the UK aiming to improve the investigation of rape and other sexual offenses. It is funded by the Home Office and applies social science research methods to inform organizational change within police forces.
